# The Role of Innate Lymphoid Cells in the Regulation of Immune Homeostasis in Sepsis-Mediated Lung Inflammation

**DOI:** 10.3390/diagnostics10100808

**Published:** 2020-10-12

**Authors:** Yuichi Akama, Naoko Satoh-Takayama, Eiji Kawamoto, Atsushi Ito, Arong Gaowa, Eun Jeong Park, Hiroshi Imai, Motomu Shimaoka

**Affiliations:** 1 Department of Molecular Pathobiology and Cell Adhesion Biology, Graduate School of Medicine, Mie University, 2-174 Edobashi, Tsu City, Mie 514-8507, Japan; a-2kawamoto@clin.medic.mie-u.ac.jp (E.K.); i-atsushi@clin.medic.mie-u.ac.jp (A.I.); arong-g@doc.medic.mie-u.ac.jp (A.G.); epark@doc.medic.mie-u.ac.jp (E.J.P.); 2 Department of Emergency and Disaster Medicine, Faculty, Graduate School of Medicine, Mie University, 2-174 Edobashi, Tsu City, Mie 514-8507, Japan; hi119@clin.medic.mie-u.ac.jp; 3 Laboratory for Intestinal Ecosystem, Center for Integrative Medical Sciences, RIKEN, 1-7-22 Suehiro-cho, Tsurumi-ku, Yokohama, Kanagawa 230-0045, Japan; naoko.satoh@riken.jp; 4 Immunobiology Laboratory, Graduate School of Medical Life Science, Yokohama City University, 1-7-29 Suehiro-cho, Tsurumi-ku, Yokohama, Kanagawa 230-0045, Japan; 5 Department of Thoracic and Cardiovascular Surgery, Faculty, Graduate School of Medicine, Mie University, 2-174 Edobashi, Tsu City, Mie 514-8507, Japan

**Keywords:** PD-1, group 2 innate lymphoid cells, sepsis, acute lung injury, IL-13, IL-33, ST2, natural and inflammatory ILC2

## Abstract

Septic shock/severe sepsis is a deregulated host immune system response to infection that leads to life-threatening organ dysfunction. Lung inflammation as a form of acute lung injury (ALI) is often induced in septic shock. Whereas macrophages and neutrophils have been implicated as the principal immune cells regulating lung inflammation, group two innate lymphoid cells (ILC2s) have recently been identified as a new player regulating immune homeostasis. ILC2 is one of the three major ILC subsets (ILC1s, ILC2s, and ILC3s) comprised of newly identified innate immune cells. These cells are characterized by their ability to rapidly produce type 2 cytokines. ILC2s are predominant resident ILCs and, thereby, have the ability to respond to signals from damaged tissues. ILC2s regulate the immune response, and ILC2-derived type 2 cytokines may exert protective roles against sepsis-induced lung injury. This focused review not only provides readers with new insights into the signaling mechanisms by which ILC2s modulate sepsis-induced lung inflammation, but also proposes ILC2 as a novel therapeutic target for sepsis-induced ALI.

## 1. Introduction

Sepsis is a life-threatening organ dysfunction caused by a dysregulated host immune system response to infection [1]. Despite advances in intensive care management and multidisciplinary treatments, the mortality rate of sepsis exceeds 20% in developed countries [2,3].

Novel types of non-T, non-B lymphocytes—termed ‘innate lymphoid cells’ (ILCs)—were discovered during the last decade. ILCs lack adaptive antigen receptors [4]. Initially, three major subsets (groups 1, 2, and 3 ILCs—ILC1s, ILC2s, and ILC3s, respectively) were defined as new immune cells that link innate and adaptive immunity according to the transcription factors required for their development and function [5]. ILC1, ILC2, and ILC3 act as the innate counterparts of Th (CD4^+^ T helper) 1, Th2, and Th17 T cell effector subsets, respectively [4]. Due to their distinct developmental cycles, they have been classified into five groups: Conventional NK (cNK), ILC1, ILC2, ILC3, and lymphoid-tissue inducer (LTi) cells [6]. The various contributions of the ILC subgroups to disease-related functions are now well documented.

This review focuses on recent advances in the understanding of the regulation and function of ILC2s in sepsis-induced lung inflammation; in particular, the balance between IL-33/ST2 and PD-L1/PD-1 signaling in ILC2s. ILC2s, the predominant subset population of ILCs in the lung [7], are characterized by secretion of the type 2 cytokines IL-5, IL-9, and IL-13. Recent studies have demonstrated that IL-33 activates ILC2s-mediated type 2 cytokines to prevent lung injury [8] and decreases the mortality of mice afflicted with Staphylococcus aureus bacteremia [9]. A more precise understanding of ILC2 functionality has revealed their critical role in executing the tissue protective effects mounted in response to various types of lung inflammation.

## 2. Innate Lymphoid Cells Protect against Pathogens and Contribute to Tissue Repair

ILCs are mainly tissue-resident cells [10] that mediate immune response in mucosal tissues. ILC1s, excluding natural killer (NK) cells, secret interferon-γ, and TNF-α, express the transcription factor T-bet [11,12]. ILC1s serve as an essential player in host immunity through the rapid secretion of an IFN-γ response to viral infections [13]. ILC2s produce type 2 cytokines and are regulated by the transcription factors GATA-3 and retinoic acid receptor-related orphan receptor alpha (RORα) [14,15]. ILC2-derived type 2 cytokines are largely responsible for the host protective immune response against helminth infection [16]. Additionally, IL-5 secreted from ILC2s in the stomach promotes bacteria-binding IgA production by plasma B cells, leading to defense against bacterial infections [17]. ILC3s and LTi cells contain the transcription factor RORγt and secret IL-17 and IL-22 [18,19,20]. ILC3-derived IL-22 protects the lungs [21] and gut [22] following bacterial infection. ILCs contribute to the biological defense against pathogens by driving the immune response.

ILCs induce protective immunity in response to infection and promote the maintenance of tissue integrity by promoting wound healing and reducing tissue damage. ILC1s contribute to damage reduction in acute liver injury by producing IFN-γ, which aids the survival of hepatocytes through the upregulation of Bcl-xL [23]. ILC2s repair intestinal epithelial cells in colitis by the production of amphiregulin (AREG), which is the epidermal growth factor-like molecule [24], following DSS treatment [25]. ILC3s facilitate skin repair by promoting epidermal re-epithelialization via the production of IL-17 [26]. These results indicate that ILC-derived cytokines play not only a protective role against pathogens, but also a regulatory role in maintaining immune homeostasis.

## 3. A Pathogenic Role of ILC2s and Lung ILC2s

ILC2s have been extensively studied in relation to allergic diseases, including asthma, which is a typical type 2 immune-mediated airway disease. Deregulated type 2 cytokine production and metabolism in ILC2s are considered to be a part of the underlying mechanisms of asthma induction [27,28,29,30]. Given recent findings showing the importance of overactive immune response by ILC2s in asthma [31,32,33], it appears that negatively regulating ILC2s may offer as a therapeutic strategy. Indeed, several already established drugs for asthma, such as β2-adrenergic agonists [34], corticosteroids [35], and leukotriene receptor antagonists [36], have been found to suppress the ILC2 immune response. Furthermore, several studies aimed to suppressing ILC2 functionality reduced airway inflammation and/or improved asthma control [37,38]. The activation of ILC2s may worsen disease progression, and research on ILC2-mediated immune response in asthma is already leading to the establishment of therapeutic strategies.

The role played by ILC2s in the lungs has been demonstrated in studies involving typical ILC2-deficient mice, such as in *Rora^sg/sg^* bone marrow transplant (BMT) mice or *Rora^fl/sg^Il7r^Cre^* mice. RORα, which is a key transcriptional factor for establishing ILC2-deficient mice, is critical to ILC2 development, although it does not substantially affect CD4^+^ T-cell development [39]. *Rora^sg/sg^* BMT mice are reconstituted by transplanting the BM of staggerer mice (*Rora^sg/sg^*) to sub-lethally irradiated lymphocyte-deficient *Rag2*^−*/*^^−^*Il2rg*^−*/*^^−^ mice [39]. Staggerer mice are naturally RORα-deficient mice and do not survive for long once past weaning. *Rora^fl/sg^Il7r^Cre^* mice are made by engineering conditionally targeted *Rora^fl/sg^* mice inter-crossed with the IL-7 receptor (IL-7R)-Cre [16]. Research findings from these representative engineered mutant ILC2-deficient mice have shown that ILC2s play a crucial role in orchestrating and mediating type 2 immunity in the lung, as indicated in Table 1. It remains to be elucidated how sepsis-induced lung inflammation affects these engineered mutant mice lacking ILC2.

## 4. ILC2s in Sepsis-Induced Lung Injury

The lung is one of the most frequently affected organs in sepsis [47], typically leading to acute lung injury (ALI). ALI secondary to sepsis is a cause of significant morbidity and mortality in septic patients (the in-hospital mortality is 60% [ALI group] vs. 14% [non-ALI group]) [48], despite efforts to improve therapy. The pathological roles of aberrantly activated lung macrophages and infiltrating neutrophils in ALI are well documented [49]; however, the mechanism of sepsis-induced lung injury remains poorly understood. ILC2s, the predominant population of ILCs in the lung [7], have garnered attention as a new player regulating type 2 immune responses. IL-5 and IL-13, representative type 2 cytokines produced by ILC2, have been shown to protect against lung injury and sepsis [50,51,52]. Additionally, ILC2-derived AREG also contributes to respiratory tissue remodeling following influenza virus-induced injury [53]. Furthermore, IL-9 is secreted as an autocrine amplifier of ILC2 function, promoting AREG production, and leading to the repair of lung tissue injuries induced by the migration of *Nippostrongylus brasiliensis* [54]. These results indicate that ILC2s maintain lung homeostasis by producing these crucial cytokines in both local and systemic inflammation. Meanwhile, an excessive ILC2 immune response can cause aggravating type 2 lung inflammation. For instance, high-mobility group box 1 promotes lung ILC2 proliferation and decreases ILC2 apoptosis following hemorrhagic shock, leading to the accumulation of ILC2 in the lung [55]. This leads to eosinophil infiltration and type 2 cytokine production in the lung, thereby contributing to lung injury following hemorrhagic shock [55]. Therefore, analyzing the immunological dynamics of ILC2s in sepsis, which cause a dysfunctional immune response to infection, could lead to new insights into the pathophysiology of sepsis-induced lung inflammation.

There are several previous studies on sepsis-induced lung inflammation involving ILC2s. IL-9-derived from ILC2s prevents lung endothelial cells from pyroptosis in cecal ligation and puncture (CLP)-induced septic lung inflammation [8]. IL-33-mediated ILC2 activation induces lung eosinophilia and reduces S. aureus-induced neutrophilia, thereby balancing dysregulated septic inflammation and reducing sepsis mortality [9]. These results indicate that ILC2s augment biological defenses by activating type 2 immune responses. In contrast, prolonged activation of ILC2s in the septic lung induces immune dysfunction. ILC2-mediated M2 macrophages produce IL-10, thereby expanding regulatory T-cell populations and mediating immunosuppression during long-term sepsis [56]. IL-33 activates ILC2s to play crucial roles [8,9,56]. Importantly, IL-33, which is released from epithelial cells in the injured lung [57], is cleaved by apoptosis-associated caspases, such as caspase-3 and -7, although not by inflammation-associated caspases, such as caspases-1, -4, and -5 [58].

These research findings indicate that lung ILC2s are crucial regulators during sepsis. In addition, dysregulated ILC2s in the immune response may be involved in the pathogenesis of sepsis-induced lung injury or mortality. How then can we control the function of ILC2s or adjust deregulated ILC2s? The molecular mechanism that balances ILC2 regulation in the septic lung is not fully understood. We have recently reported important research findings that address this question.

## 5. ILC2 Subsets in the Septic Lung

**Figure 1:**
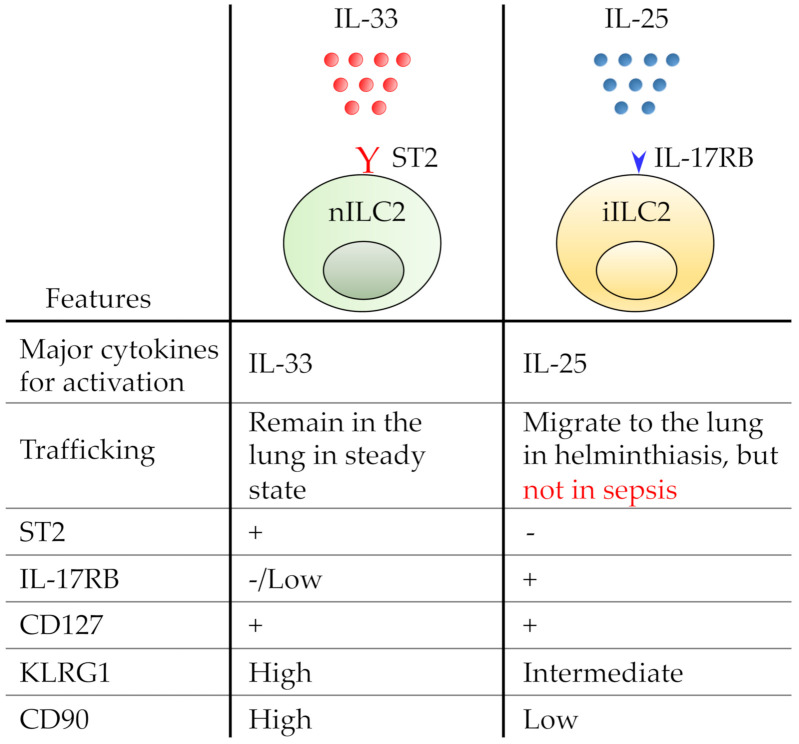
Representative features of natural group two innate lymphoid cell (nILC2) and inflammatory ILC2 (iILC2). nILC2 expresses ST2 (red Y), a receptor of IL-33. iILC2 expresses IL-17RB (blue arrowhead), a receptor of IL-25.

Two ILC2 subsets have been reported in the lung: Natural ILC2 (nILC2) and inflammatory ILC2 (iILC2) (Figure 1) [59]. nILC2s are characterized by Lineage (Lin)^−^ ST2^+^ IL17RB^−/lo^ CD127^+^ KLRG1^hi^ CD90^hi^ [59,60,61]. nILC2 subset is comprised of tissue-resident ILC2s [10] and is activated by IL-33 [59]. On the other hand, iILC2 subset, which is characterized by Lin^-^ ST2^−^ IL17RB^+^ CD127^+^ KLRG1^int^ CD90^lo^ [59,60,61], is thought to move from the gut to the lung in response to either IL-25 administration or to helminth infections [60]. Interestingly, we hardly observed any significant increase in the number of iILC2s; indeed, levels of IL-25 mRNA expression over the seven days following CLP surgery in our recent study remained very low [62]. These research findings possibly indicate that iILC2s hardly migrate to the septic lungs, and that their role therein is limited. Therefore, in this review, we focused on the role of nILC2s.

## 6. The Mechanism That Drives IL-33/ST2 Signaling Stimulates ILC2s

IL-33, which stimulates ILC2s and promotes IL-5 and IL-13 production [14], is predominantly expressed by damaged epithelial cells in the lung following inflammation (i.e., recruited neutrophils release proteases and oxygen-derived free radicals [63]). ST2 (also known as IL1RL1) is a receptor of IL-33 and is also expressed on a variety of immune cells, such as Th2 cells [64], regulatory T cells [65], mast cells [66], M2 macrophages [67], and eosinophils [68]. ST2 serves as a marker of murine lung ILC2s [7]. The expression levels of ST2 on ILC2s differs based on where the latter is located [69]. It is upregulated by TGF-β signaling via the MEK-dependent pathway [70].

IL-33 binds a heterodimer formed by ST2 and the IL-1 receptor accessory protein (IL-1RAP). This signaling induces the recruitment of myeloid differentiation primary response protein 88 (MyD88), which is located in the cytoplasmic region of ST2. Subsequently, MyD88 recruits IL-1R-associated kinase 1 (IRAK1), IRAK4, and TNF receptor-associated factor 6 (TRAF6), resulting in the activation of either the NF-κB or AP-1 pathway [71]. The signal stimulates lung ILC2s and promotes IL-5 and IL-13 production. The molecular mechanism underlying IL-33/ST2 signaling in ILC2 [72,73] may share some similarities with Th2 cells [74] in mediating MyD88. However, a recent study revealed that this signaling helps to promote Foxp3 and GATA3 expression in colonic Tregs [65]. IL-33/ST2 signaling may express different functions in a variety of pathways. Its molecular mechanism is not yet fully understood.

## 7. The Mechanism Underlying PD-1/PD-L1 Signaling Inhibits ILC2s

Programmed death-ligand 1 (PD-L1), a ligand of programmed death 1 (PD-1) also known as B7 homolog 1 or B7-H1, is widely expressed in a variety of cells including activated macrophages [75]. The expression level of PD-L1 is enhanced by pro-inflammatory cytokines, including TNF-α, and IFN-γ, and IL-10 [76]. Lung ILC2s also express PD-L1 and promote Th2 polarization by binding to PD-1 on CD4^+^ T cells [77].

PD-1 is expressed on lymphocytes, such as T cells and B cells. PD-1 is not expressed on resting T cells but is expressed after activation [78]. Signaling through TCR or BCR upregulates PD-1 on lymphocytes [79]. PD-1 expression in ILC2s is increased by IL-33 stimulation [80].

The inhibitory mechanism and function of PD-1/PD-L1 signaling may differ depending on cell types. For instance, the signal in T cells can inhibit T-cell functions by recruiting SHP-2, thereby dephosphorylating their downstream signaling molecules within the PI3K/AKT and MAPK/ERK signaling pathways. These pathways are triggered by both T-cell receptors (TCRs) and CD28 [76]. PD-1 of ILC2s restricts their numbers and functions through the inhibition of STAT5 signaling [81]. PD-1/PD-L1 signaling has been shown to negatively regulate both ILC2s and T cells; however, the details of this molecular mechanism are not fully understood.

The interaction of PD-1 and PD-L1 is well documented as a cause of impaired T-cell functionality. Blocking this signaling has been established as a successful therapy for several cancers by ameliorating T-cell exhaustion. Since cancer shares several immunosuppressive mechanisms with sepsis, blocking PD-1/PD-L1 signaling on T cells has been studied as a therapeutic target during sepsis [82,83]. Interestingly, our recent study demonstrated that PD-1 levels on both ILC2s and PD-L1 in the lungs are upregulated during sepsis. This prompted us to evaluate whether ILC2s are a part of the pathogenesis underlying sepsis-induced lung inflammation by PD-1-mediated inhibition of the down-regulation of immune responses, and whether blocking PD-1/PD-L1 signaling in ILC2s represents a potential treatment target.

## 8. The Functional Dynamics of Lung ILC2s during Sepsis

**Figure 2:**
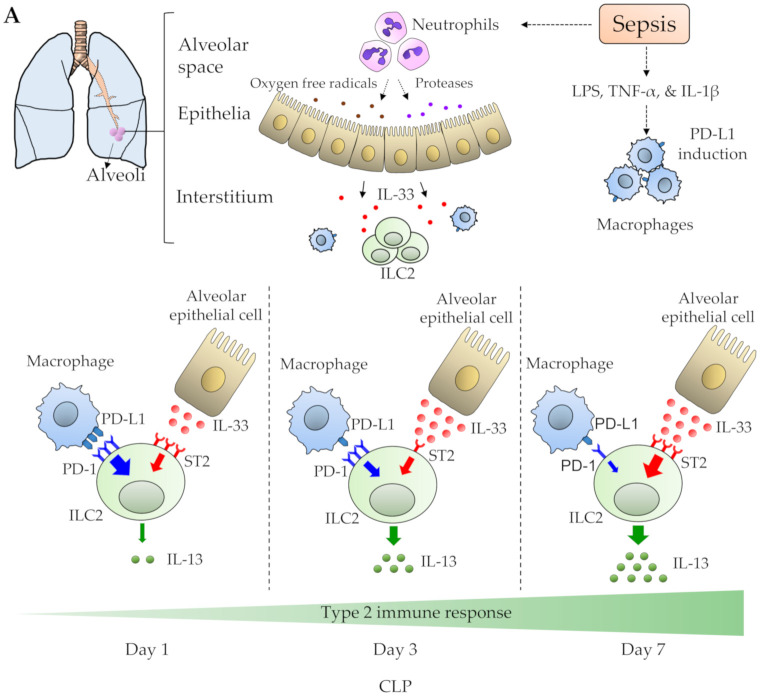

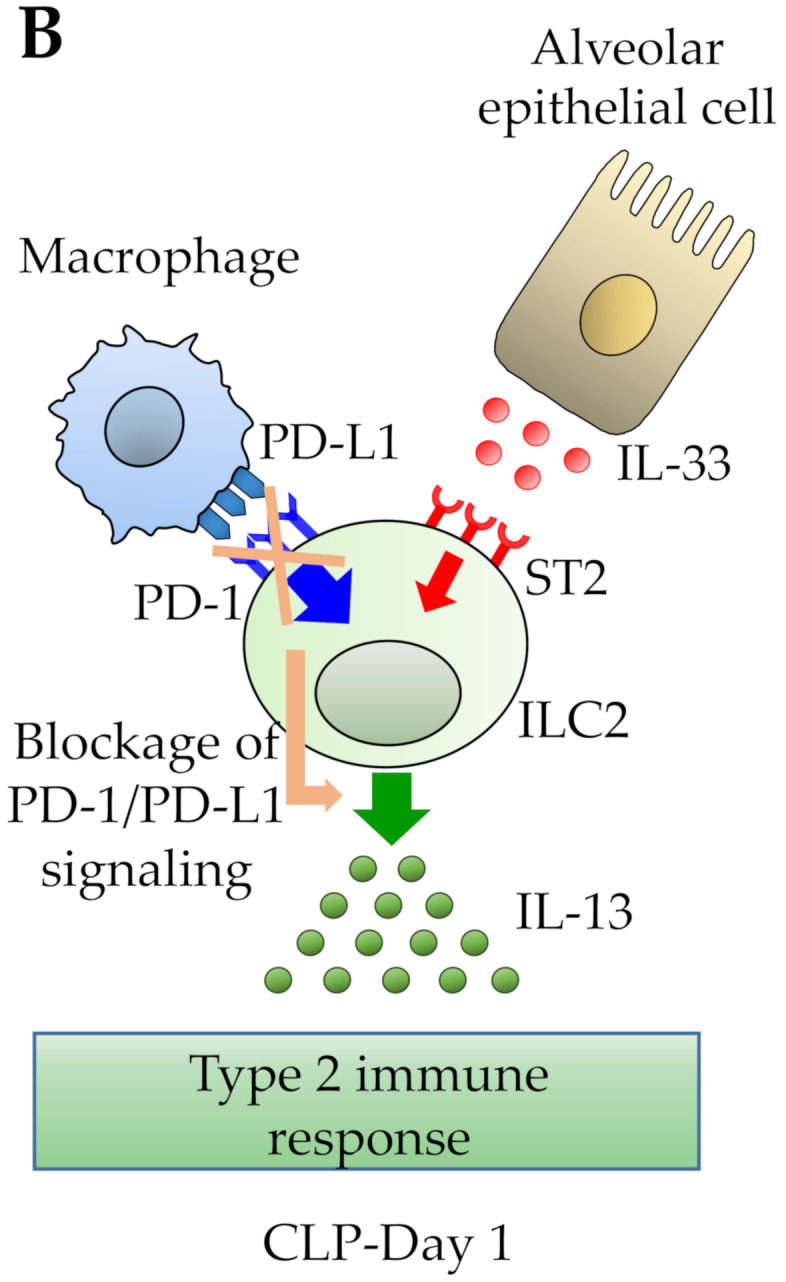
Schematic representation of a proposed model for (**A**) the balance between the ST2-IL-33 axis and the PD-1-PD-L1 axis, and (**B**) a novel therapeutic strategy for the septic lung by blocking PD-1/PD-L1 signaling in ILC2s. (**A**) IL-33 released from damaged lung epithelial cells binds to ST2, leading to NF-κ B and AP-1 activation, which promotes IL-13 production in ILC2s (ST2-IL-33 axis; red arrows). In contrast, the PD-L1 upregulated in macrophages elicits PD-1-mediated inhibitory signals related to STAT5 inactivation. This inhibits IL-13 production in ILC2s (PD-1-PD-L1 axis; blue arrows). The balance of signaling strengths between the ST2-IL-33 axis and the PD-1-PD-L1 axis could determine the levels of IL-13 production in ILC2s. (**B**) Blocking PD-1/PD-L1 signaling in ILC2s may induce IL-13 secretion, leading to impaired lung inflammation during sepsis. CLP: cecal ligation and puncture, PD-1: programmed death 1, PD-L1: Programmed death-ligand 1

Both IL-33/ST2 signaling and PD-L1/PD-1 signaling are essential to fulfilling the functions of ILC2s. Indeed, they affect the balance of ILC2 regulation in the septic lung. However, there is little research on the functional dynamics of lung ILC2s over the different time points, early to late phase, during sepsis. We investigated the transitions of IL-33/ST2 signaling and PD-L1/PD-1 signaling in the septic lung, as well as IL-13 production in ILC2s [62]. During days 1 through 7 after CLP, we discovered that the balance of signaling strengths between the IL-33/ST2 and PD-L1/PD-1 pathways affected the levels of IL-13 production in ILC2s in the septic lung (Figure 2A). In short, IL-13 production by ILC2s in the lung was initially inhibited by sepsis, but then gradually increased. Although IL-33/ST2 signaling in ILC2s was robust, IL-13 secretion remained low during the early phase of sepsis. This might be explained by the high PD-1 expression evident on ILC2s.

We also evaluated IL-5 secretion in ILC2s [62]. IL-5 production levels were not significantly different during our experimental period. Our results suggest that IL-5 production was only slightly more influenced than that of IL-13 in the septic lung. There is no information on the expression levels of other ILC2-producible cytokines, such as IL-9 or AREG, although the biological defense mechanism associated with ILC2s may be perturbed by PD-1 in sepsis.

IL-33 certainly affects the dynamics of ST2 and PD-1 expression on ILC2s. Our experiments evaluating both ST2 and PD-1 expression levels of ILC2s after CLP in IL-33 knockout mice showed that the changes in expression levels during days 1 through 7 after CLP were smaller than those of wild-type mice [62]. This result is consistent with the fact that IL-33 stimulation upregulates the expression levels of PD-1 on ILC2s [80]. As TGF-β upregulates ST2 expression on ILC2s [70], it could have an effect on ILC2s during sepsis. Other sepsis-induced cytokines may also change these expression levels; however, the details surrounding this phenomenon remain unclear.

## 9. PD-1 on ILC2s as a Target for PD-1 Blocking Therapy in the Septic Lung

Clinically proven pharmacological interventions to alleviate ALI are currently lacking [49]. A few non-pharmacological supportive treatments, such as the conservative fluid strategy to prevent lung edema formation and lung-protective mechanical ventilation, have shown some effectiveness for ALI [49,84,85,86]; nevertheless, establishing a novel pharmacological therapeutic strategy is urgently needed.

In mice, IL-13 has been shown to exert a protective role during sepsis by reducing inflammation. Blocking IL-13 in a CLP-induced sepsis mouse model revealed worse rates of mortality and lung injury, which are associated with increased neutrophil-activating chemokine and proinflammatory cytokine levels in the lungs [87]. Furthermore, ILC2-derived IL-13 polarizes alveolar macrophages to M2 macrophages in one in vitro experiment [88]. A recent study has shown that by adaptively transferring M2 macrophages into lung-injured mice, lung inflammation and damage can be significantly reduced [89]. This indicates that a rapid shifting to M2 macrophages may regulate lung inflammation and damage. In our study, sepsis simultaneously induced low levels of IL-13 production and high levels of PD-1 expression on ILC2s during the early phase of sepsis (CLP day 1). Therefore, blocking PD-1/PD-L1 signaling may lead to increased IL-13 production and the development of new therapeutic strategies for sepsis-induced acute lung injuries (Figure 2B).

In fact, blocking PD-1/PD-L1 signaling has been shown to improve outcomes in CLP-induced sepsis mice [90,91]. The effectiveness of blocking PD-1/PD-L1 signaling has been proven in animal experiments studying sepsis, which is thought to occur by ameliorating T-cell exhaustion [82]. Additionally, our results may indicate that blocking PD-1/PD-L1 signaling has beneficial effects; e.g., relieving the inhibition of IL-13 secretion on ILC2s. On the other hand, since the role of PD-1/PD-L1 signaling in septic lung ILC2s remains unclear, the impact on already existing protective mechanisms through AREG or IL-9 production, or the side effects of blocking PD-1/PD-L1 signaling, such as the potential for excessive IL-13 production leading to the immunosuppression or inhibition of Th2 polarization, is uncertain.

Furthermore, the roles played by IL-13 in sepsis also remain unclear. Although the effects of developmental roles of IL-13 in the remodeling of the immune system need to be considered, IL-13 deficient mice have experienced survival benefits, along with decreased tissue damage following CLP [92]. Our concept requires further investigation; however, our research findings provide new insights into the role of ILC2s in the pathophysiology of sepsis-induced lung inflammation, particularly regarding the possibility of inhibiting IL-13 production in ILC2s by PD-1.

## 10. Summary and Future Challenges

ILCs are increasingly recognized to play key roles in the immune response to sepsis. ILCs help to induce a protective response against infection and also promote the maintenance of tissue integrity by ameliorating tissue damage and/or repairing it. We have summarized in the present report the roles and molecular mechanisms of the ST2-IL-33 axis and PD-1-PD-L1 axis in ILC2s. Although ILC2s are not regulated by IL-33/ST2 and PD-1/PD-L1 signaling alone, these signaling mechanisms are crucial regulators for ILC2s in sepsis-induced lung inflammation. Additionally, we discussed how IL-13 production is regulated between the two signaling systems, and the potential therapeutic strategies of activating PD-1-mediated IL-13 production on ILC2 in the septic lung. Further investigations should be conducted regarding how lung ILC2s are activated and controlled under different settings and how they interact with other immune cells through PD-1/PD-L1 binding and its molecular mechanisms. ILC2s may be the last piece of the puzzle for deciphering the complicated immune responses mounted against human sepsis-induced acute lung injuries, potentially leading to novel therapeutic strategies.

## Figures and Tables

**Table 1:** List of previous reports that studied type 2 immunity in the lung of representative engineered mutant ILC2-deficient mice.

***Rora^sg/sg^* BMT Mice**		
**Disease Model**	**Roles of ILC2s in the Lung**	**References**
Papain-induced asthma model	ILC2s play a crucial role in the inflammatory response to allergens, even in the presence of Th2 cells.	[39]
Papain-induced asthma model	ILC2-derived IL-13 primes naive T cells to differentiate into Th2 cells by promoting the migration of DCs to LNs.	[40]
House dust mite-induced asthma model	BAL eosinophils are significantly decreased in ILC2-deficient mice.	[41]
Papain-induced asthma model	ILC2s induce DCs, thereby promoting memory Th2 function.	[42]
Hemorrhagic shock model	ILC2-derived IL-5 promotes IL-5 production of neutrophils.	[43]
Helminth-infected mouse model	ILC2-deficiency leads to a reduction in eosinophil accumulation.	[44]
***Rora^fl/sg^Il7r^Cre^* Mice**		
**Disease Model**	**Roles of ILC2s in the Lung**	**References**
Papain-induced asthma model	ILC2s induce DCs, thereby promoting memory Th2 functionality. (The authors used both *Rora^sg/sg^* BMT and *Rora^fl/sg^Il7r^Cre^* mice).	[42]
Papain-induced asthma model Helminth-infected mouse model	ILC2s promote increases in Th2 cells in mLN and IgE concentrations in the lung.	[45]
Cigarette smoke exposure model	ILC2s help induce collagen deposition following cigarette exposure.	[46]

ILC2s: group two innate lymphoid cells, DCs: dendritic cells, LNs: lymph nodes, BAL: bronchoalveolar lavage.
